# Silsesquioxane Cages under Solvent Regimen: The Influence of the Solvent on the Hydrolysis and Condensation of Alkoxysilane

**DOI:** 10.1021/acs.inorgchem.4c00460

**Published:** 2024-05-08

**Authors:** Anna Władyczyn, Łukasz John

**Affiliations:** Faculty of Chemistry, University of Wrocław, 14 F. Joliot-Curie, 50-383 Wrocław, Poland

## Abstract

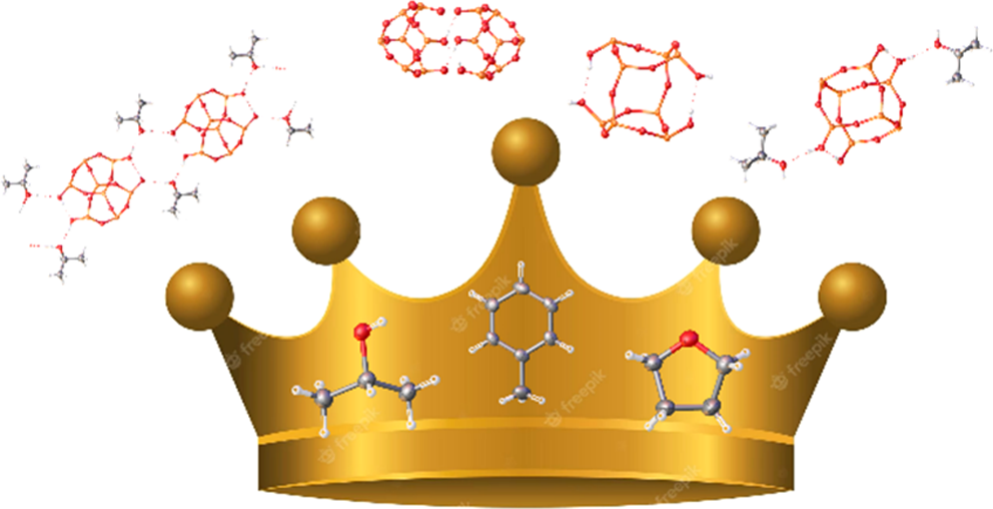

This study investigates the formation mechanisms of oligomeric phenyl silanols, focusing on polyhedral oligomeric silsesquioxane (POSS) and double-decker silsesquioxane (DDSQ) derivatives. Combining literature reports and crystal structures of solvated derivatives obtained in our laboratory, we show that the solvent choice significantly influences their structures. POSS-based silanols prefer aprotic solvents like THF, preserving dimerization, while double-deckers form stable architectures in protic solvents like isopropanol. This discrepancy arises from different stabilization mechanisms. Our findings enhance our understanding of hydrolytic condensation involving trimethoxyphenylsilane and suggest aprotic solvents for efficient reactions with POSS-based silanols.

## Abstract 2

The substrate’s environment matters—it turns out that complicated hydrolytic condensation of trimethoxyphenylsilane leads to the formation of silsesquioxane cages of different architectures depending on the solvent used.

## Introduction

The commercial utilization of organic silane derivatives was initiated in the 1950s.^1^ These silanes, which feature hydrolyzable groups like alkoxy and functionalized organic substituents, were extensively applied as “cross-linkers”^2^ and “coupling agents”.^3^ The intricate processes of hydrolysis and condensation that these silanes undergo are recognized as simultaneous and complex equilibria, influenced by various factors such as substituent type, pH level, concentration, temperature, solvent, catalyst, and reaction time.^4−8^ The mechanistic details remain unclear given the many variables impacting the reaction outcome. Insufficiently detailed knowledge leads to silica-based materials being synthesized without comprehensively comprehending the fundamental reaction kinetics. Consequently, optimizing these procedures becomes laborious, typically relying on a time-intensive “trial and error” approach. In the early stages of research on silsesquioxanes chemistry, Gilkey et al.^9^ developed a method to produce fully condensed silsesquioxanes with various substituents like *n*-propyl, *n*-butyl, ethyl, methyl, cyclohexyl, 2-methyl pentyl, and phenyl. This approach resulted in mixtures containing cages with differing silicon atom counts (*T*_3_, *T*_6_, *T*_8_, and *T*_12_) and polymers. On the other hand, Brown et al.,^10^ while examining the polymerization of different trifunctional silicone systems, found that after hydrolyzing and condensing trichloro cyclohexyl silsesquioxane in acetone, the resulting mixture possessed a significant crystalline fraction—primarily 84% cyclohexyl-T_2_(OH)_4_ dimer. In another study, the same research group^11^ observed that the hydrolytic condensation of trichlorophenylsilane, in the presence of KOH and solvents like toluene or bis(2-methoxyethyl) ether at their boiling points, yielded mainly a prepolymer (94%) along with *T*_8_ and *T*_12_ species (4–5%). Contrarily, using solvents like benzene, nitrobenzene, benzyl alcohol, or pyridine led to the formation of the T_8_ derivative, while THF predominantly produced a T_12_ compound. Solvents such as acetonitrile or diethylene glycol dimethyl ether resulted in an insoluble polymeric gel. Additionally, Rebrov et al.,^12^ while conducting hydrolytic condensation with hexafunctional branched organotetrasiloxanes, unexpectedly obtained only the T_8_ cage structure instead of the anticipated T_4_, with a yield of 23%. In turn, Unno et al.^13^ reported on the synthesis of cyclohexyl-T_6_ derivative, employing silanetriols or 1,1,3,3-tetrahydroxydisiloxane and dicyclohexylcarbodiimide (DCC) as a dehydration agent, but this method failed when applied to T_8_-type cages. Moreover, Hurkes et al.^14^ examined Bassindale’s^15^ protocol and demonstrated that T_8_ cages can be synthesized from trisilanetriols and disiloxane-1,1,3,3-tetrols with high yield, using tetrabutylammonium fluoride (TBAF) as a fluoride anion source. This outcome suggests that the hypervalency of silicon in the intermediate product plays a critical role in the successful condensation process.

**Scheme 1:**
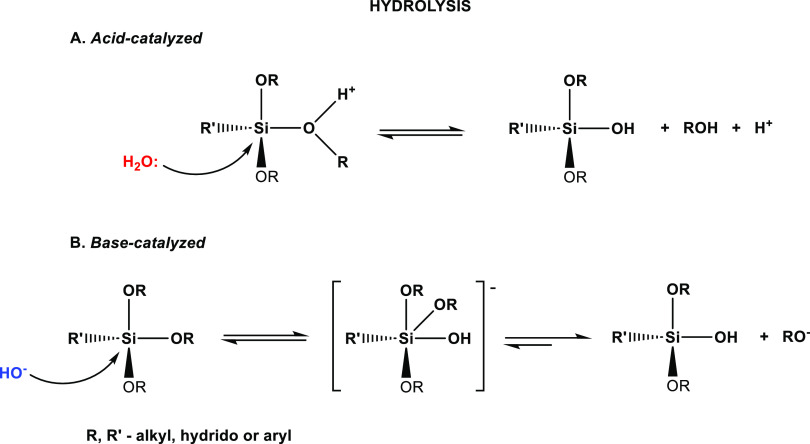
Hydrolysis of Trialkoxysilane under Acidic and Basic Conditions

Trialkoxysilanes play a pivotal role as substrates in the synthesis of silsesquioxanes,^16^ silicone resins,^17^ and in surface modification^18^ through sol–gel reactions.^19^ Regrettably, unlike tetralkoxysilanes,^20^ their reactivity remains inadequately comprehended and described to date. It is widely recognized that the polycondensation process of silanes initiates with hydrolysis, followed by condensation, resulting in the formation of a sol, gel, or silsesquioxanes. These reactions are dynamic equilibria that transpire in competition.^21−24^Scheme 1 illustrates the reaction equations for hydrolysis (acid- and base-catalyzed) of trialkoxysilanes. Proposedly, under acidic hydrolysis conditions (Scheme 1A), the alkoxide group is initially protonated, inducing the withdrawal of the electron density from the silicon atom. This renders the silicon atom more electrophilic and susceptible to attack by water molecules. Conversely, water molecules dissociate under basic conditions (Scheme 1B), generating nucleophilic hydroxyl anions that engage with the silicon atom. Significantly, the hydrolysis steps exhibit gradual deceleration in acidic conditions and acceleration in basic conditions, leading to substantial divergences in the resulting product structure. Basic conditions foster the creation of small, highly branched agglomerates, forming a colloidal sol with weak interparticle connections. On the other hand, acidic conditions yield a sol with a chain-like structure.^24^ However, the complexity deepens when considering that the hydrolysis and condensation reactions are accompanied by concurrent re-esterification and depolymerization reactions.^25^

**Scheme 2:**
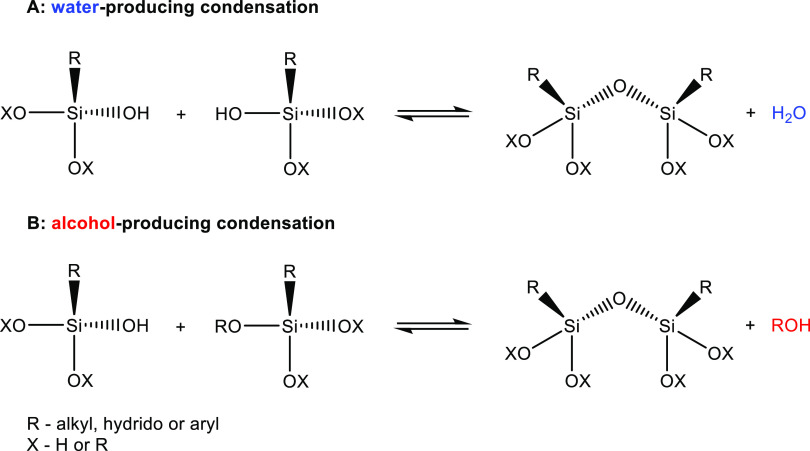
Reactions That May Occur after Hydrolysis: Condensation of the Silanol Groups with the Formation of Water (A) and Reaction between the Silanol Group and the Alkoxy Group Leading to the Formation of Alcohol (B)

The formation of siloxane bonds through condensation can transpire via either water (Scheme 2A) or alcohol (Scheme 2B) production. Certainly, oligomeric silsesquioxanes unquestionably stand out as the most captivating outcomes of the sol–gel reaction, encompassing their architectural intricacy, properties, and a promising range of applications.

The general formula of silsesquioxanes is represented as (RSiO_1.5_)_*n*_, wherein R stands for a nonreactive organic group or a hydrogen atom, and *n* = 4, 6, 8, 10, 12.^26^ The initial report of synthesizing oligomeric silsesquioxanes dates back to 1955, as reported by Sprung and Guenther.^27^

**Scheme 3:**
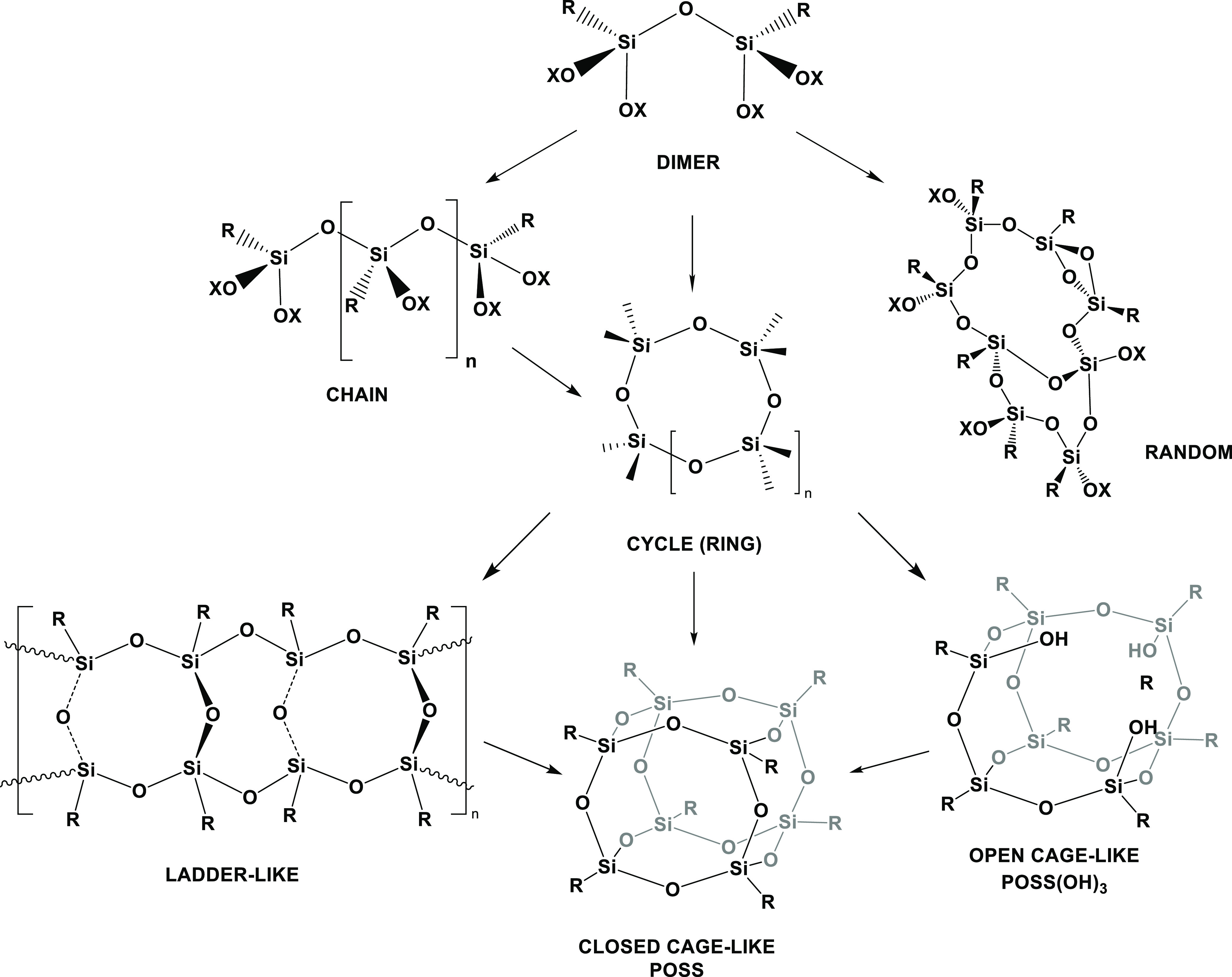
Hydrolytic Condensation Sequence Led to Silsesquioxanes Forming with Different Architectures

They observed the emergence of small quantities of white precipitates during the polymerization reactions of alkyltriethoxysilanes. Within the realm of silsesquioxanes, diverse architectures manifest, including cycles, randomly branched structures, ladder-like configurations, perfect ladder structures, and both open and closed cages (POSS) (Scheme 3).

To date, it has been determined that due to the diminished steric hindrance exhibited by the silanol group (Si–OH) compared to the alkoxy group (Si–OR), the pace of hydrolysis accelerates correspondingly with the increase in the count of alkoxide groups that undergo hydrolysis. The substituent’s size also influences the process’s pace; the hydrolysis rate of alkoxysilanes diminishes with an increase in the steric bulk of the alkoxide group. It is striking that silanols exhibit noteworthy acidity, surpassing even that of alcohols (p*K*_a_ = 18) or water (p*K*_a_ = 15.6). Consequently, nucleophilic silanolates (Si–O−) are generated in alkaline settings—these groups are vital for the mechanism driving the creation of silsesquioxanes under the base-catalyzed conditions of the condensation phase.^27^ Nevertheless, the precise cause behind the selective formation of oligomeric cages with specific shapes or sizes (such as the number of SiO_1.5_ subunits in the oligomer) remains incompletely comprehended. Endeavors aimed at acquiring derivatives like POSS and DDSQ have drawn our attention to a case of silane reactivity that could potentially aid in unraveling this enigma.

**Scheme 4:**
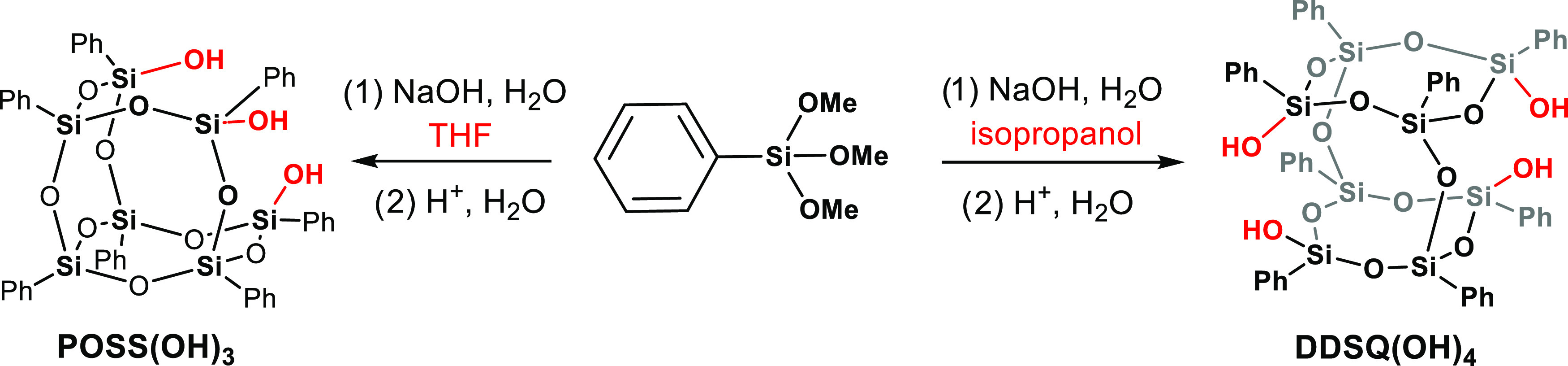
Reactivity of Trimethoxyphenylsilane in Different Solvents Leading to the Formation of **POSS(OH)**_**3**_ and **DDSQ(OH)**_**4**_

A specific instance of reactivity involving trimethoxyphenylsilane has piqued our curiosity. Brown^28^ made a groundbreaking discovery regarding the mechanisms of phenylsilanetriol, revealing its tendency to selectively undergo condensation, predominantly forming a tetraol unit in acetone. Performed studies demonstrated that the destiny of the resulting precursors, derived from this tetraol, is influenced by the conditions of the reaction, such as the solvent type, duration, and temperature. In this research, Brown also revised his earlier belief, proposed five years ago,^29^ that the condensation product forms a “ladder structure”. Furthermore, more than a decade after this study was published, Frye and Klosowski raised doubts about its findings in a correspondence to *the Journal of the American Chemical Society* editor.^30^ They challenged Brown’s conclusion that the polymer formed was a ladder-like structure, suggesting instead that it comprised open polycyclic cages. This hypothesis was based on their observations of the behavior of reactive, intermediate phenylsilanetriols, which they argued were more likely to form randomly linked aggregates rather than the orderly, ladder-like architectures previously suggested. This particular substrate serves a dual purpose; it is utilized for producing both the open cage polyhedral oligomeric silsesquioxane (**POSS(OH)**_**3**_) and functions as a precursor for crafting the double-decker silsesquioxane (**DDSQ(OH)**_**4**_) as depicted in Scheme 4. Intriguingly, the published protocols detailing the methodologies for attaining these open cages exhibit slight disparities in the stoichiometric ratio of silane/NaOH/water and, more notably, in the choice of solvent. In light of this, we posit that the solvent could wield a pivotal influence on the final product’s structure engendered through the hydrolysis and condensation processes of trimethoxyphenylsilane.

A synthesis of **DDSQ(OH)**_**4**_ was described for the first time in a patent application by Chisso Corporation in 2004.^31^ According to this disclosure, the procedure occurs within an inert environment, employing isopropanol as both the solvent and substrate alongside silane, NaOH, and H_2_O, with a ratio of 1/1.15/0.66. The resulting mixture was stirred while refluxing for 4 h and left at room temperature for 24 h. The initial product, forming as a sodium adduct, precipitates after the reaction, permitting separation from the mixture via filtration. In a subsequent step, neutralization was required, often employing agents like acetic acid^32^ to achieve the silsesquioxane containing four silanol groups with a notably high yield (98,^33^ 90,^34^ and 86%^35^).

**Scheme 5:**
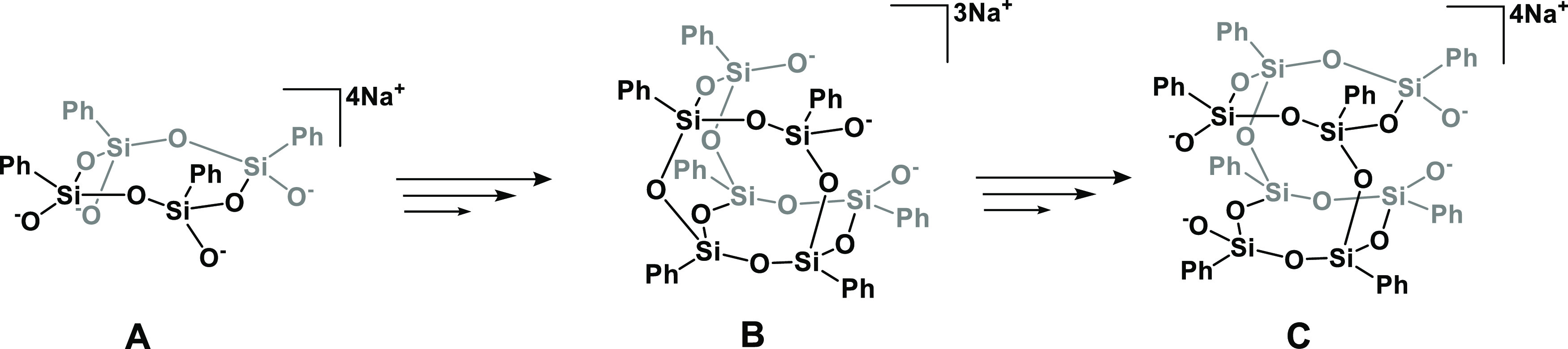
Probable Trialkoxyphenylsilane Condensation Path Sym-*cis*-tetraphenylsilsesquioxane (A), **POSS(ONa)**_**3**_ (B), and **DDSQ(ONa)**_**4**_ (C).

Nevertheless, none of the studies mentioned above provide insights regarding the byproducts arising from the synthesis. The solubility of the sodium adduct in organic solvents is exceedingly poor, leading to a lack of spectroscopic evidence confirming the product’s purity. The qualitative assessment of these systems can be undertaken only after their interaction with halogenosilanes. Ervithayasuporn^36^ uncovered that a secondary product is generated in this reaction, specifically an open cage **POSS(OH)**_**3**_. However, the authors note that this product constitutes a mere 9% of the final mixture. Kawakami et al.^32^ conducted a comprehensive investigation into the condensation mechanism of phenyltrialkoxysilane. The central objective of this study was to scrutinize the reaction products meticulously (utilizing MALDI-TOF MS and ^29^Si NMR techniques) for various stoichiometric ratios of the employed substrates. Of paramount significance, samples were collected at intervals from the reaction’s initiation (up to 70 h), revealing that **POSS(OH)**_**3**_ serves as an intermediate product along the formation of **DDSQ(OH)**_**4**_ (Scheme 5).

Furthermore, the interpretation of the findings permits us to infer that sym-*cis*-tetraphenylsilsesquioxane (Scheme 5A) is likely generated in the initial phase of condensation, subsequently undergoing conversion into **POSS(ONa)**_**3**_ (Scheme 5B) possibly through condensation involving triphenylsilsesquioxane (T_3_) or three phenylsilane molecules. Eventually, reorganization and further condensation processes culminate in the formation of **DDSQ(ONa)**_**4**_ (Scheme 5C). Intriguingly, in isopropanol, **POSS(OH)**_**3**_ manifests as an unstable intermediate on the pathway toward **DDSQ(OH)**_**4**_. This observation further implies that the open cage structural configuration remains independent of variations in the reactant ratios.

The same research group embarked on an exploration of the intricate hydrolytic condensation process. In the subsequent studies released a year later, the authors presented an alternative approach to synthesizing open cage **POSS(OH)**_**3**_ and **DDSQ(OH)**_**4**_ structures.^37^ Instead of initiating the synthesis of silsesquioxanes from trimethoxyphenylsilane, the octaphenyl cage (T_8_) was hydrolyzed. Hydrolysis was conducted using NaOH and water with isopropanol as the solvent. The analytical methodology employed for assessing the reaction products mirrored that described in the previous study^32^—the postreaction mixture containing sodium adducts was subjected to a reaction with trimethylsilane to enhance the solubility of derivatives within organic solvents. This facilitated the execution of product characterization via MALDI–TOF MS and ^29^Si NMR measurements. The authors formulated a hypothesis suggesting that “open” cages could be formed during alkaline hydrolysis through two potential mechanisms: (i) hydrolysis of Si–O–Si bonds and detachment of smaller silanol fragments; (ii) hydrolysis leading to the “cleaving” of the cage at specific bond sites, subsequently undergoing reorganization through condensation into open cages. To validate this hypothesis, an experiment was devised involving the hydrolysis of a mixture of T_8_ POSSs with varying substituents, such as phenyl, *o*-methylphenyl, and deuterated phenyl substituents. If the hydrolysis aligned with the first strategy, then the reaction products should be open cages with identical substituents. Contrariwise, open cages with mixed substituents would be anticipated if the second hypothesis held. The analysis outcomes on the postreaction mixture unequivocally demonstrated that the reaction products indeed consisted of cages with mixed substituents, thereby corroborating the validity of the second hypothesis. Furthermore, within the product mixture, **POSS(ONa)**_**3**_ and **DDSQ(ONa)**_**4**_ were detected. In overextended reaction durations, the proportion of **DDSQ(OH)**_**4**_ within the postreaction mixture increased, corroborating that **POSS(ONa)**_**3**_ displayed less stability in isopropanol. Consequently, the hydrolysis of condensed *octa*-substituted POSSs culminates, and the subsequent phase involves self-assembling “open” cages through condensation. Hence, the hypothesis regarding the selectivity of the corner-opening process, which aimed to yield heterosubstituted POSSs, was disproven,^38^ and is still under investigation.^100^

Inferences can be drawn that the hydrolysis process occurs in a stochastic manner. Yet, intriguingly, a propensity toward the condensation of **POSS(ONa)**_**3**_ and **DDSQ(ONa)**_**4**_ systems emerges despite **DDSQ(OH)**_**4**_ exhibiting superior stability in isopropanol. This hypothesis finds reinforcement in the fact that the procedure outlined for obtaining **POSS(OH)**_**3**_, as outlined by Ohno,^39^ involves the utilization of THF as a solvent, distinct from isopropanol. Furthermore, certain researchers attest to a noteworthy 98% yield^33^ from the reaction, a remarkable feat considering the 9% efficiency observed with isopropanol as the solvent.^36^ The stoichiometric ratios of the reactants (silane/NaOH/H_2_O) engaged in this reaction stand at 1:0.44:1.26. Nonetheless, it remains plausible that these proportions could be chosen arbitrarily. The observations presented by Kawakami^32^ indicate that the ratio of reactants is not paramount, as the initially generated **POSS(ONa)**_**3**_ progressively transitions into the more stable **DDSQ(OH)**_**4**_ over time, particularly in an isopropanol medium.

At this juncture, it is essential to note that most silanol derivatives display instability due to the potential for spontaneous intermolecular self-condensation.^40^ Research has substantiated that the stability of aryl silanetriols hinges on the substitution pattern within the phenyl substituent.^41^ Instances of the slightest effective condensation have been observed with silanes possessing significantly hindered steric profiles in their phenyl substituents. This suggests that sterically demanding substituents might incline toward silanol hydrogen bonding rather than condensation. Sprung^42^ also presented supporting evidence for the aforementioned hypothesis, demonstrating that bulky substituents enable the isolation of substantial quantities of low-molecular-weight products from partial hydrolysis. These substituents also stabilize the silanol functions in these products, preventing further condensation. This reasoning possibly elucidates why **POSS(OH)**_**3**_ and **DDSQ(OH)**_**4**_ do not undergo self-condensation. The inclination of silanols to engage in hydrogen bonding becomes more pronounced with the acidity of the specific silanol unit.^41^ Various studies utilizing IR spectroscopy and titration techniques have established that the acidities follow a sequence of arylsilanols > alkylsilanols > arylcarbinols > alkylcarbinols.^43^ Guided by this established hierarchy, it is reasonable to anticipate the formation of robust intermolecular or intramolecular hydrogen bonds within open phenyl-substituted cages. The exploration of dimer versus monomer formation in cage structures is a fascinating topic. Brown et al.^28^ initially observed that POSS-trisilanol is capable of forming stable dimers. Subsequently, Pietschnig et al.,^40^ through ab initio calculations, found that for both heptamethyl-POSS(OH)_3_ and heptaisobutyl-POSS(OH)_3_, dimer formation is energetically more favorable. Specifically, they noted that heptamethyl-POSS(OH)_3_ shows a stability increase of 24.3 kcal mol^–1^ over two separate monomers, while for heptaisobutyl-POSS(OH)_3_, this stability gain is 16.3 kcal mol^–1^ less. They proposed that the variance in the stabilization energy might be due to the size of the substituents, indicating that larger substituents potentially weaken intermolecular hydrogen bonding. However, the role of solvents in influencing dimer formation was highlighted by Unno et al.,^44^ when ^1^H NMR dilution and Fourier transform infrared (FTIR) spectroscopy were used to study hydrogen bonding in different types of incompletely condensed silsesquioxanes (POSS-triol, POSS-diol, and POSS-mono-ol with isobutyl substituents). The solvents used were CDCl_3_, C_6_D_6_, DMSO-*d*_6_, and acetonitrile-*d*_3_. They discovered that in DMSO, POSS-triol does not form dimers due to the strong hydrogen bond it forms with the DMSO molecule. This suggests that stronger intermolecular hydrogen bonds might occur in less polar solvents such as chloroform or toluene. Furthermore, they observed that as the concentration of POSS-triol decreased, the dimer dissociated into monomers, evidenced by the OH signal shifting toward the high field in the ^1^H NMR spectra. This implies that in less polar environments, stronger intermolecular hydrogen bonds might form. This solvent dependence was also observed by Pietschnig et al.^45^ They presented silanetriol derivatives in which different structural motifs based on the solvent used for crystallization can be realized for the same substituents. It turns out that the dimeric structure of 2,6-dimesitylphenylsilanetriol is obtained when the compound is recrystallized from apolar solvents, such as cyclohexane or benzene. However, when crystallization occurs in polar THF, the molecules adopt a linear, tubular arrangement.

Considering the above-mentioned principles and the literature discrepancies, we resolved to explore the impact of solvents on cage architecture. This exploration is grounded in theoretical deliberations and is substantiated through empirical analysis. The experimental facet rests on insights derived from X-ray assessments of single crystals that constitute solvated formations. Drawing upon the pertinent crystal structures, we formulated a hypothesis suggesting that the crux of open cage stabilization lies in the potential for intermolecular hydrogen bond formation. This stays in agreement with other examined structures, as these intermolecular hydrogen bonds could occur between the silanol groups of silsesquioxanes and solvents.

## Experimental Section

### Materials

All chemicals (phenyltrimethoxysilane, 97%; NaOH) and solvents were purchased from commercial sources (ABCR, Sigma-Aldrich, Merck) and used without further purification. The **DDSQ(OH)**_**4**_ and **POSS(OH)**_**3**_ were prepared as reported previously with yields of 58 and 80%, respectively.^32,46^ Single crystals of **DDSQ(OH)**_**4**_ and **POSS(OH)**_**3**_ solvates were obtained by the slow evaporation of toluene (for **DDSQ(OH)**_**4**_·**toluene**) and isopropanol (for **DDSQ(OH)**_**4**_·**2(isopropanol)** and **mono-POSS(OH)**_**3**_).

### Crystallography

Crystallographic data of **mono-POSS(OH)**_**3**_ were obtained using an Xcalibur, Ruby, Gemini, ultra-diffractometer K by using a fine-focus sealed Mo–Kα tube, λ = 0.71073 Å. The crystal was kept at 210.15 K during the data collection.

Crystallographic data of **DDSQ(OH)**_**4**_·**toluene** as well as for **DDSQ(OH)**_**4**_·**2(isopropanol)** were obtained using an XtaLAB Synergy R, DW system, HyPix-Arc 150 diffractometer by using graphite-monochromatized Cu Kα radiation (λ = 1.54184 Å) at 100 K. Frame integration, data reduction, and absorption corrections were performed using the CrysAlisPro^47^ program package. Using Olex^2^ software,^48^ structures were solved with the SHELXS^49^ structure solution program using Direct Methods and refined with the SHELXL^50^ refinement package using least squares minimization. For each compound, the positions of the hydrogen atoms on the silanol groups (SiOH) were found in the difference Fourier maps and were initially refined isotropically. Other H atom positions were idealized by the HFIX command. The drawing of the model of crystal structures was made with Olex^2^ software.^48^ Details of the crystal parameters, data collection, and refinement for structures are listed in Table 1. Crystallographic data for the structures of **mono-POSS(OH)**_**3**_, **DDSQ(OH)**_**4**_·**toluene**, and **DDSQ(OH)**_**4**_·**2(isopropanol)** reported in this paper have been deposited with the Cambridge Crystallographic Data Centre as supplementary publication nos. CCDC 2201339, 2193846, and 2193848, respectively. Copies of the data can be obtained free of charge by application to CCDC, 12 Union Road, Cambridge CB2 1EZ, UK [fax.: (Internet.) + 44 1223/336–033; e-mail: deposit@ccdc.cam.ac.uk].

**Table 1:** Experimental Details for **mono-POSS(OH)**_**3**_, **DDSQ(OH)**_**4**_·**toluene**, and **DDSQ(OH)**_**4**_·**2(isopropanol)**

	mono-POSS(OH)_3_	DDSQ(OH)_4_·toluene	DDSQ(OH)_4_·2(isopropanol)
deposition number	2201339	2193846	2193848
empirical formula	C_45_H_46_O_13_Si_7_	C_48_H_44_O_14_Si_8_	C_48_H_44_O_14_Si_8_·2(C_3_H_7_OH)
formula weight	991.45	1069.55	1189.74
temperature/K	210.15	100	100
crystal system	monoclinic	monoclinic	triclinic
space group	*P*2_1_/*n*	*P*2_1_/*n*	*P*1̅
*a*/Å	15.109(7)	17.177(6)	10.753(17)
*b*/Å	13.108(3)	8.520(3)	11.54(5)
*c*/Å	25.366(5)	19.964(5)	13.752(7)
α/°	90	90	72.712(2)
β/°	103.81(3)	106.410(2)	82.1490(16)
γ/°	90	90	64.6500(19)
volume/Å^3^	4878(3)	2802.7(12)	1472(7)
*Z*	4	2	1
ρ_calc_g/cm^3^	1.350	1.267	1.342
μ/mm^–1^	0.257	2.309	2.276
*F*(000)	2072.0	1112.0	624.0
crystal size/mm^3^	0.452 × 0.258 × 0.173	0.341 × 0.232 × 0.149	0.219 × 0.193 × 0.143
radiation	Mo Kα (λ = 0.71073)	Cu Kα (λ = 1.54184)	Cu Kα (λ = 1.54184)
2θ range for data collection/°	6.532–61.512	6.006–151.092	6.732–151.208
index ranges	–21 ≤ *h* ≤ 21, –14 ≤ *k* ≤ 17, –36 ≤ *l* ≤ 20	–21 ≤ *h* ≤ 21, –6 ≤ *k* ≤ 10, –24 ≤ *l* ≤ 24	–13 ≤ *h* ≤ 10, –14 ≤ *k* ≤ 14, –17 ≤ *l* ≤ 17
reflections collected	28 915	26 871	24 624
independent reflections	13 258 [*R*_int_ = 0.0241, *R*_sigma_ = 0.0387]	5731 [*R*_int_ = 0.0234, *R*_sigma_ = 0.0197]	5988 [*R*_int_ = 0.0225, *R*_sigma_ = 0.0218]
data/restraints/parameters	13258/93/606	5731/36/399	5988/0/472
goodness-of-fit on *F*^2^	1.387	1.055	1.108
final *R* indexes [*I* ≥ 2σ (*I*)]	*R*_1_ = 0.0743, *wR*_2_ = 0.1984	*R*_1_ = 0.0301, *wR*_2_ = 0.0818	*R*_1_ = 0.0347, *wR*_2_ = 0.0957
final *R* indexes [all data]	*R*_1_ = 0.1129, *wR*_2_ = 0.2154	*R*_1_ = 0.0323, *wR*_2_ = 0.0832	*R*_1_ = 0.0382, *wR*_2_ = 0.0978
largest diff. peak/hole/e Å^–3^	1.06/–0.42	0.32/–0.35	0.34/–0.58

## Results and Discussion

### Solvents’ Influence on Silsesquioxane Cage Architecture

Taking into account the premises mentioned in the Introduction section and inaccuracies, we decided to investigate how solvents influence the architecture of cages. Given the disparity in the resulting product structures, we examined two categories of solvents: tetrahydrofuran (THF) and isopropanol.

**Figure 1:**
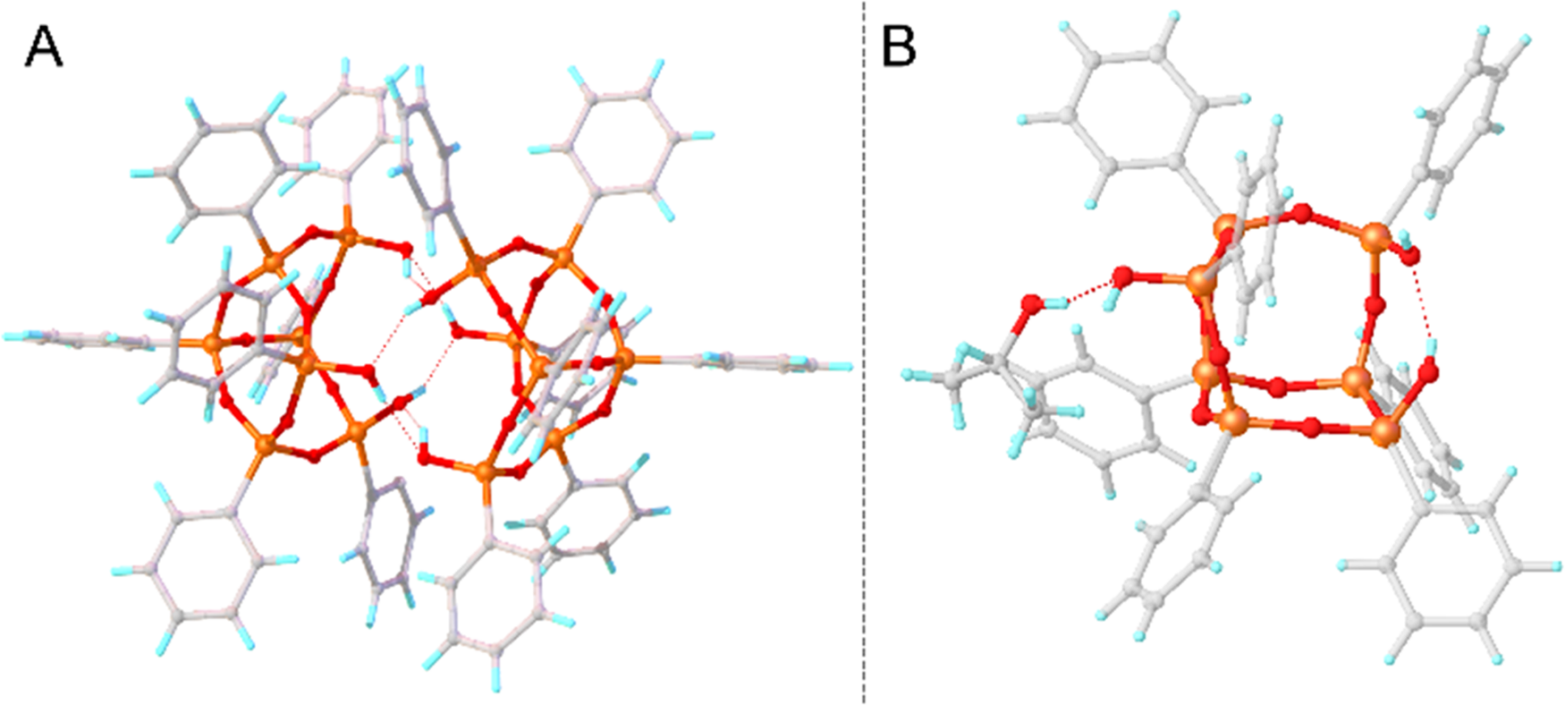
Molecular structure of **POSS(OH)**_**3**_ as a dimer (A)^46^ and **POSS(OH)**_**3**_ as a monomer (B). Color code: gray—carbon, orange—silicon, red—oxygen, blue—hydrogen.

Kawakami^46^ was the first to unveil the crystal structure of phenylic **POSS(OH)**_**3**_ (Figure 1A). This derivative crystallized from a mixture of aprotic solvents, chloroform/hexane, adopting a monoclinic space group (*P*1̅) as a dimer (**dimer-POSS(OH)**_**3**_). This dimeric form is brought forth through cooperative intramolecular hydrogen bonding and embodies the *C*_i_ symmetry. Six hydrogen bonds are in symmetry equivalent positions (with atoms positioned at a special position—inversion center), manifest with O–H···O distances of 2.035(2), 2.162(3), and 1.977(6) Å. The angles characterizing the cyclic system of hydrogen bonds are 171.77(1), 158.10(6), and 145.95(3)°. Analysis of the experimental data points to these interactions being categorized as moderately strong, predominantly electrostatic hydrogen bonds.^51^ In a study highlighting the catalytic activity of **POSS(OH)**_**3**_, Jagannathan^52^ also disclosed the crystal structure of this derivative, which lacked any solvent in the lattice. These structures exhibit similar crystal lattice parameters and share identical space groups. Notably, the structure presented by Jagannathan et al. records a lower *R*-factor (3.45% as opposed to 6.51%). The authors, guided by the kinetics of the reaction, observed that beyond a concentration of 25 mM, **POSS(OH)**_**3**_ exists in the solution in dimeric form, validated by both crystal structure analysis and results from the VTNA kinetic study protocol. These findings also suggest that **POSS(OH)**_**3**_ exists as a monomer below this concentration threshold. Notably, this analysis was also carried out within an aprotic solvent (CD_2_Cl_2_). For comparative purposes, the structures of heptaisobutyl-POSS(OH)_3_^44^ and heptacyclohexyl-POSS(OH)_3_^53^ were also included in the crystal database. These compounds similarly exhibit dimer formation, suggesting this as characteristic behavior. Furthermore, in the study by Unno et al.,^44^ which investigated the dimer–monomer equilibrium concerning solvent type and concentration, the dimerization constant was determined. This constant was found to be *K*_dim_ = 174 mol^–1^·dm^3^ in CDCl_3_ and 1075 mol^–1^·dm^3^ in C_6_H_6_.

Nonetheless, the crystal structure of the monomeric form of **POSS(OH)**_**3**_ (**mono-POSS(OH)**_**3**_) is unveiled here for the first time (Figure 1B). This derivative was acquired utilizing the methodology previously reported by Kawakami.^46^

### Isopropanol as a Cage Separator

The monomeric structure of **POSS(OH)**_**3**_ emerges within a monoclinic system (*P*2_1_/n space group). Crystalline formations were procured through gradual evaporation from a saturated solution of isopropanol. Detailed crystal data and refinement parameters are outlined in Table 1. It is noteworthy that endeavors to derive a crystal structure from a THF solution yielded unsuccessful outcomes. Instead of **mono-POSS(OH)**_**3**_, the octaphenyl cage, which is a small amount of the product impurity, crystallized. This structure assumes the form of an isopropanol solvate. Within the asymmetric unit, a single molecule resides, while within a unit cell, four molecules are generated through symmetry operations: (i) 1/2 – *x*; (ii) 1/2 – *y*; (iii) 1/2 – *z*. For each **mono-POSS(OH)**_**3**_ molecule, an accompanying isopropyl alcohol molecule engages in a hydrogen bond interaction. Intriguingly, isopropanol’s interaction is limited to only one of the three silanol groups. This phenomenon mirrors the observation of Feher,^53^ who deduced that one among the three silanol groups consistently exhibits heightened reactivity. This particular silanol group’s distinctive behavior could likely be attributed to robust intramolecular hydrogen bonding, which in turn lowers the p*K*_a_ of the silanol group’s hydrogen, enhancing its acidity. The average intermolecular O–H···O bond length within **dimer-POSS(OH)**_**3**_ registers at 2.058(2) Å. Conversely, within **mono-POSS(OH)**_**3**_, the intramolecular O–H···O bond extends to 1.966(3) Å, while the intermolecular (cage-solvent) O–H···O bond spans 1.860(7) Å. These bond lengths could indicate that the forces governing cage–cage interactions are less potent compared with cage-isopropanol interactions. An important synthetic implication stemming from this revelation pertains to the potential utilization of polar, protic solvents (such as alcohols) as separators for cages. This maneuver could heighten their reactivity in corner-capping reactions involving trialkoxysilanes. In line with Kawakami’s^46^ observations, the phenyl cage of **POSS(OH)**_**3**_ does not undergo corner-capping with 3-aminopropyltriethoxysilane within an acetone medium in the presence of triethylamine (contrary to the less sterically demanding isobutyl cage). Should the root of this scenario lie in a pronounced inclination toward dimerization, employing a separating solvent might potentially elevate the reaction yields.

The crystal structure depicted in Figure 1B can also provide insight into the rationale behind performing the synthesis of **POSS(OH)**_**3**_ in THF^46^ and not, like **DDSQ(OH)**_**4**_, in isopropanol.^32^ Evidence from prior research^40,54^ has substantiated that the structure of **POSS(OH)**_**3**_ is bolstered by the potential for dimerization (as supported by density functional theory (DFT) calculations). As corroborated by the crystal structure, the hydrogen interaction with isopropyl alcohol holds greater strength, consequently inhibiting the formation of energetically advantageous dimers. Including isopropanol within the crystal lattice of **DDSQ(OH)**_**4**_, another partially condensed silsesquioxane, offers insights into the mechanisms behind stabilizing these derivatives. The synthesis of **DDSQ(OH)**_**4**_ followed a well-established protocol with a 58% yield.^30^

**Figure 2:**
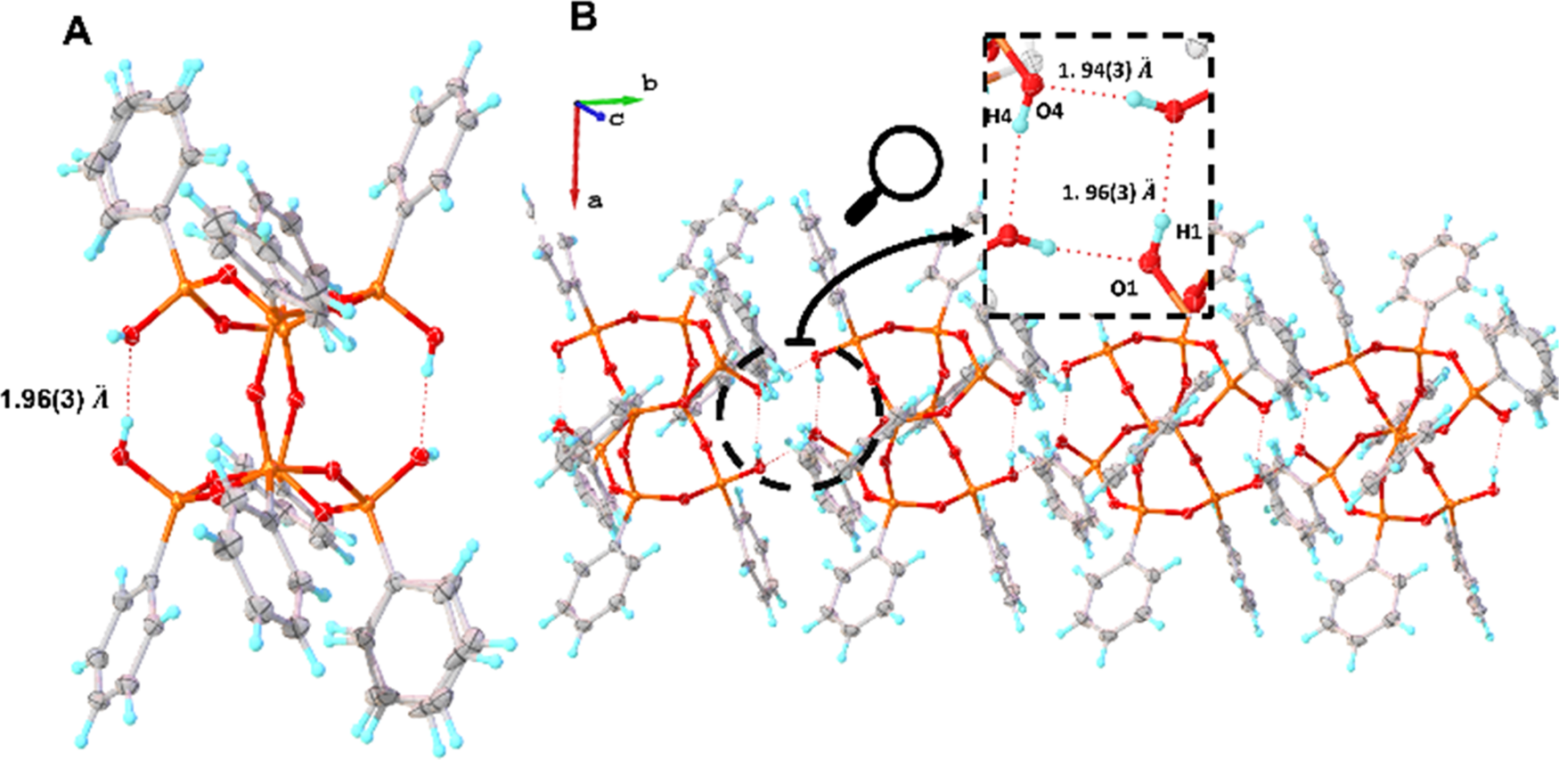
Crystal structure of **DDSQ(OH)**_**4**_·**toluene** (A) and fragment of the crystal packing (B). Color code: gray—carbon, orange—silicon, red—oxygen, blue—hydrogen.

Initially, we succeeded in procuring the **DDSQ(OH)**_**4**_·**toluene** solvate (owing to the substantial solvent molecule disorder; it was subsequently extracted using the SQUEEZE^55^ procedure). These crystals were obtained through gradual evaporation from a saturated isopropanol solution. A detailed account of crystal data and refinement parameters can be found in Table 1. The ultimate *R*_1_ parameter stands at 3.01%. The structure crystallized within a monoclinic system (*P*2_1_/*n* space group). Half of a molecule exists within the asymmetric unit, while two molecules emerge within a unit cell. The molecule depicted in Figure 2A possesses *C*_i_ symmetry. Within the **DDSQ(OH)**_**4**_·**toluene** entity, a corresponding toluene molecule is present in the unit cell, albeit in a highly disordered state.

Given toluene’s aprotic nature, it abstains from hydrogen-bonding interactions with the silanol groups. Instead, the structure’s stabilization likely hinges upon π-stacking interactions between toluene and the phenyl rings of the cages. The arrangement of molecules within the crystal lattice leads us to presume that **DDSQ(OH)**_**4**_ molecules derive their stability from the potential for intra- and intermolecular hydrogen bond formation, as depicted in Figure 2A,B. The intramolecular O–H···O bond length encompasses 1.96(3) Å, whereas the intramolecular variant measures 1.94(3) Å. Correspondingly, the associated angles stand at 170(3) and 172(3)°. These data collectively suggest that this hydrogen interaction can likewise be categorized as moderately strong, predominantly electrostatic hydrogen bonds.^51^

### Isopropanol as a Linker

**Figure 3:**
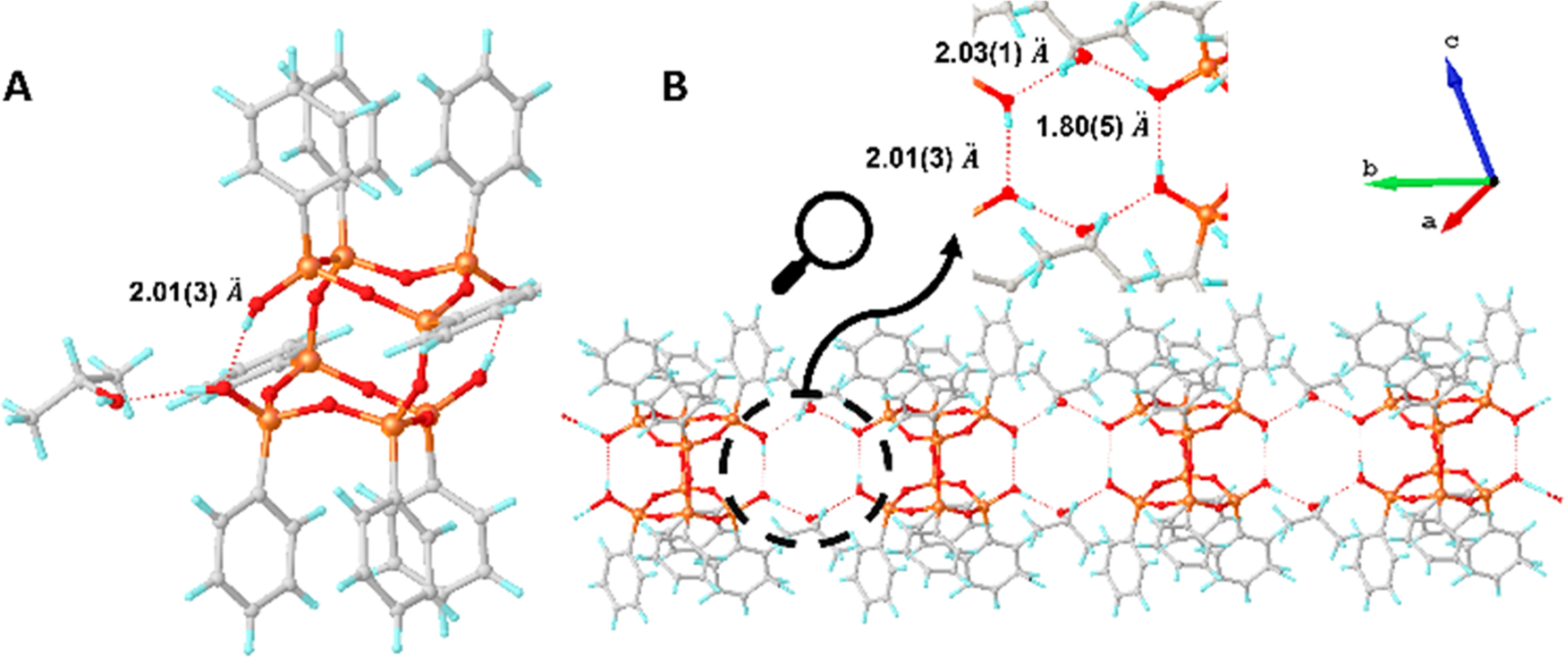
Crystal structure of **DDSQ(OH)**_**4**_·**2(isopropanol)** (A) and a fragment of the crystal packing (B). Color code: gray—carbon, orange—silicon, red—oxygen, blue—hydrogen.

An intriguing augmentation to the considerations emerges from the insights garnered through the scrutiny of another solvate, **DDSQ(OH)**_**4**_·**2(isopropanol)** (Figure 3A,B). The structure occurs within the triclinic system, embracing the *P*1̅ space group, thus adopting *C*_i_ symmetry. The ultimate *R*_1_ parameter culminates at 3.47%. Within the asymmetric unit, half of a molecule resides alongside one isopropanol molecule. Symmetry operations, underpinned by an inversion center, bestow the unit cell with one **DDSQ(OH)**_**4**_ molecule and two isopropanol molecules. For a more comprehensive exposition of crystal data and refinement parameters, refer to Table 1.

The structural analysis provides evidence that the **DDSQ(OH)**_**4**_ derivative is stabilized due to the possibility of forming linear chains through hydrogen bonds. They can exist between **DDSQ(OH)**_**4**_ molecules, as in the case of **DDSQ(OH)**_**4**_·**toluene**, or between molecules of a protic solvent that can simultaneously act as a hydrogen bond donor and acceptor (such as for isopropanol in this case). The intramolecular O–H···O bond length is 2.01(3) Å, while intramolecular are 2.03(1) and 1.80(5) Å. The angles are, respectively, 174(4), 170(3), and 171(3)°. These experimental values suggest this hydrogen interaction can also be classified as moderate-strong, mostly electrostatic hydrogen bonds.^51^ However, it is significant that the length of the **DDSQ(OH)**_**4**_—isopropanol bond is shorter (1.80(5) Å) than the length of the **DDSQ(OH)**_**4**_**-DDSQ(OH)**_**4**_ (2.01(3) Å) bond. This difference, although small, may suggest that the strength of the DDSQ interaction with solvent molecules is greater than the hydrogen interaction inside the cages.

## Conclusions

In summary, this paper aims to delve into the intricate mechanism underlying the formation of oligomeric phenyl silanols, explicitly focusing on **POSS(OH)**_**3**_ and **DDSQ(OH)**_**4**_. Integrating theoretical insights with experimental support through crystal structures of solvated forms of these derivatives constitutes the central approach. Considering the significant role of hydrogen bonds in stabilizing the structure of oligomeric silanols, we advance the notion that the choice of solvent exerts a pivotal influence on the resultant silanol geometry. Examination of the crystal structures reveals a preference for **POSS(OH)**_**3**_ formation in an aprotic solvent environment such as THF. Aprotic solvents do not compete with silanol groups for hydrogen bonding, thereby preserving dimerization.

In contrast, **DDSQ(OH)**_**4**_ appears more inclined to form in a protic solvent, like isopropanol. This discrepancy can be attributed to two factors: (i) the stabilization of **POSS(OH)**_**3**_ stemming from the potential for intermolecular dimer formation (**POSS(OH)**_**3**_**-POSS(OH)**_**3**_) and (ii) the stability of **DDSQ(OH)**_**4**_ arising from the prospect of creating linear polymeric chains through intermolecular hydrogen bonds. Notably, the research demonstrates the capacity of protic solvents to bolster the creation of such chains. With these findings, we anticipate a more coherent comprehension of the perplexing hydrolytic condensation mechanism involving trimethoxyphenylsilane. Additionally, our study underscores the potential for enhanced efficiency in the corner-capping reaction with **POSS(OH)**_**3**_ using aprotic solvents.

Our research could significantly impact the efficient synthesis of well-defined POSS-based phenyl silanol derivatives. As extensively documented in the literature, these materials are crucial for their superior solubility and exceptional thermal stability.^56−60^ With our findings, there is a promising opportunity to produce these derivatives selectively and efficiently, potentially leading to a wide range of new applications in material science.
